# Near-Complete Genome Sequence of a Human Pegivirus Variant Isolated from a Hepatitis E Virus-Infected Patient

**DOI:** 10.1128/MRA.01151-18

**Published:** 2019-01-24

**Authors:** Xiaodan Zhang, Wang Li, Chenglin Zhou

**Affiliations:** a Zhenjiang Center for Disease Prevention and Control, Zhenjiang, Jiangsu, China; b Clinical Laboratory Center, Taizhou People’s Hospital, Taizhou, Jiangsu, China; KU Leuven

## Abstract

Using viral metagenomics, we analyzed the virome of a blood sample from a hepatitis E patient. We identified viral sequences showing significant similarity to human pegivirus (HPgV), anellovirus, and hepatitis E virus. The near-complete genome of a genotype 3 HPgV strain was acquired and characterized.

## Abstract (precis)

Using viral metagenomics, we analyzed the virome of a blood sample from a hepatitis E patient. We identified viral sequences showing significant similarity to human pegivirus (HPgV), anellovirus, and hepatitis E virus.

## ANNOUNCEMENT

Human pegivirus (HPgV) is an RNA virus belonging to the recently proposed Pegivirus genus in the *Flaviviridae* family (1), which can be further grouped into 7 genotypes with distinct geographical distributions (2, 3). Transmission of HPgV through blood and sexual exposure has been well documented. Following infection, most people clear their viremia, concomitantly developing antibodies, but the virus can also cause chronic infection in an estimated 20 to 30% of infected people (4). An HPgV prevalence of 1 to 4% has been noted among blood donors in developed countries (5).

In February 2016, a 56-year-old female patient was admitted to the Affiliated Hospital of Jiangsu University complaining of general fatigue for more than 1 month. On admission, laboratory studies revealed an alanine aminotransferase level of 602 U/liter and an aspartate aminotransferase level of 766 U/liter (normal, <50 U/liter). Testing for anti-hepatitis E virus (anti-HEV) antibodies and HEV RNA revealed that IgM was positive, but no IgG or HEV RNA could be detected (6). The serum sample was then subjected to virus nucleic acid detection using viral metagenomics. Written informed consent for the publication of clinical details was obtained from the patient.

Library preparation and computational analysis were performed as previously described (7, 8). Briefly, the serum sample was filtered through a 0.45-mm filter (Millipore) to remove eukaryotic and bacterial cell-sized particles and then was subjected to nuclease enzymes to reduce the concentration of free nucleic acids (8). The remaining total nucleic acid was then extracted using the QIAamp mini viral RNA kit (Qiagen). The extracted nucleic acid was subjected to reverse transcription reactions using reverse transcriptase (SuperScript IV; Invitrogen) and random hexamer primer, followed by a single round of DNA synthesis using Klenow fragment polymerase (New England BioLabs). A library was then constructed using the Nextera XT DNA sample preparation kit (Illumina) and sequenced on the MiSeq Illumina platform, which generated a total of 57,416 250-bp-long paired-end reads for this particular sample. Bioinformatics analysis was performed according to previous studies (9, 10). During bioinformatics analysis, the reads which were identical in their first 5 to 55 bp were considered identical, and only one random copy of duplicates was kept. The cleaned reads were *de novo* assembled within each barcode by SOAP*denovo*2 version r240 using a k-mer size of 63, with default settings. The assembled contigs, along with unassembled reads, were aligned to the viral proteome database using BLASTx, with an E value cutoff of <10^−5^. Candidate viral hits were then compared to an in-house nonvirus nonredundant protein database to remove false-positive viral hits. Contigs without significant BLASTx similarity to the viral proteome database were searched against viral protein families in the vFam database (11) using HMMER3 (12–14) to detect remote viral protein similarities.

The results indicated that 2,584 sequence reads showed significant sequence similarity to the viruses, including, in order of sequence read abundance, HPgV (2,565 reads), anellovirus (15 reads), and hepatitis E virus (4 reads). The other nonviral sequence reads mostly showed similarity to human, bacterial, and bacteriophage genomes. The 2,565 HPgV reads could be assembled into an 8,923-bp contig, which showed 91% nucleotide sequence identity with an HPgV strain (GenBank accession no. U94695) isolated from a hepatitis patient in China in 1997 (15). The nearly complete genome was then acquired by primer walking based on the genome of accession no. U94695 and Sanger sequencing; it was found to be 9,157 bp in length and was named zj-180501. zj-180501 encodes a 2,842-amino acid (aa) polyprotein, showing 99% amino acid sequence similarity to the polyprotein of an HPgV strain (GenBank accession no. D87263) isolated from a Japanese patient (16).

**FIG 1:**
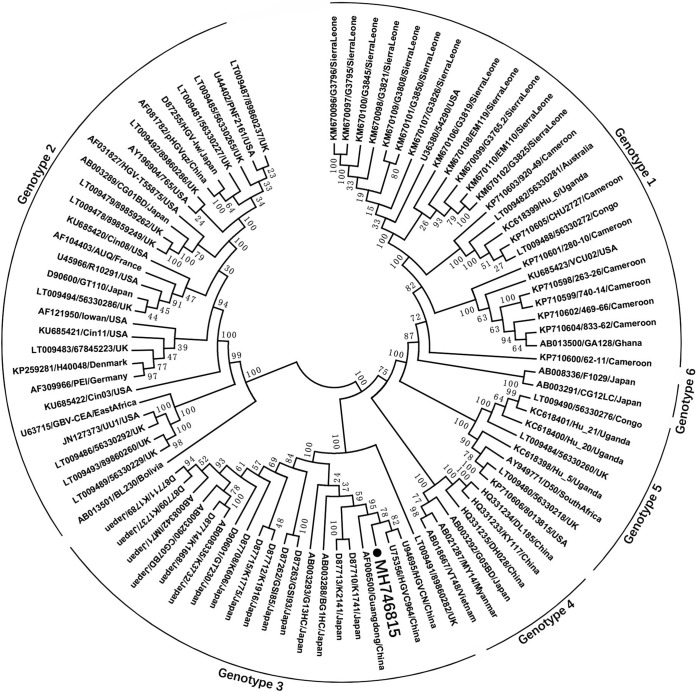
Phylogenetic analysis based on the complete genomes of zj-180501 and 91 other representative HPgVs. Multiple-sequence alignment was performed in the ClustalW program. The phylogenetic tree was constructed using the neighbor-joining method and evaluated using the interior branch test method with the MEGA 7 software. Percent bootstrap support is indicated at each node. The GenBank accession number, strain name, and country of isolation for the reference genomes are given. The HPgV strain identified in this study is marked by a dot.

Phylogenetic analysis based on the nearly complete genomes of zj-180501 and 91 other representative HPgVs indicated that zj-180501 belonged to genotype 3, clustering closely with the Chinese HPgV strain with the accession no. U94695, sharing 91.3% nucleotide sequence identity (Fig. 1). Using the Recombination Detection Program (RDP) (17), recombination analysis based on the 107 complete genomes of HPgV strains available in GenBank was performed, which showed no detectable recombinant sites in zj-180501.

### Data availability.

The raw sequence reads were deposited in the Sequence Read Archive with accession no. SRR7691636. The genomic sequences of HPgV zj-180501 were deposited in GenBank under accession no. MH746815.
